# Evaluation of Scopoletin from *Penicillium janthinellum* Snef1650 for the Control of *Heterodera glycines* in Soybean

**DOI:** 10.3390/life11111143

**Published:** 2021-10-26

**Authors:** Jichen Yan, Zhifu Xing, Piao Lei, Aatika Sikandar, Ruowei Yang, Yuanyuan Wang, Xiaofeng Zhu, Xiaoyu Liu, Haiyan Fan, Yuanhu Xuan, Lijie Chen, Yuxi Duan

**Affiliations:** 1 Nematology Institute of Northern China, Shenyang Agricultural University, Shenyang 110866, China; yjc891013@163.com (J.Y.); zfxing1994@163.com (Z.X.); piaolei9411@163.com (P.L.); aatika1122@gmail.com (A.S.); yangrw2021@163.com (R.Y.); wyuanyuan1225@syau.edu.cn (Y.W.); syxf2000@syau.edu.cn (X.Z.); xwlkitty@syau.edu.cn (X.L.); fanhaiyan2017@syau.edu.cn (H.F.); xuanyuanhu115@syau.edu.cn (Y.X.); chenlj-0210@syau.edu.cn (L.C.); 2 College of Plant Protection, Shenyang Agricultural University, Shenyang 110866, China; 3 College of Biological Science and Technology, Shenyang Agricultural University, Shenyang 110866, China; 4 College of Science, Shenyang Agricultural University, Shenyang 110866, China

**Keywords:** soybean, *Penicillium janthinellum*, scopoletin, seed coating, *Heterodera glycines*, biological agent

## Abstract

Soybean cyst nematode (SCN) (*Heterodera glycines* Ichinohe) is responsible for causing a major soybean disease globally. The fungal strain *Penicillium janthinellum* Snef1650 was evaluated against *H. glycines*. However, the effective determinants of the *P. janthinellum* strain are unknown. By performing pot experiments, a functioning compound was isolated from *P. janthinellum* Snef1650 through organic solvent extraction, semi-preparative HPLC, Sephadex LH-20 column chromatography, and silica gel column chromatography, and the isolated compound was identified to be scopoletin through 1H NMR, 13C NMR, and HPLC–MS. The pot experiments indicated that the treatment of soybean seeds with scopoletin drastically reduced the SCN population. The field experiments performed in 2017 and 2018 revealed that scopoletin decreased over 43.7% juveniles in the roots and over 61.55% cysts in the soil. Scopoletin treatment also promoted soybean growth and improved its yield, with an increase in plot yield by >5.33%. Scopoletin obtained from *P. janthinellum* Snef1650 could be used as an anti-*H. glycines* biocontrol agent.

## 1. Introduction

Soybean cyst nematode (SCN) (*Heterodera glycines* Ichinohe) infects soybean (*Glycine max*) plantations and hence causes significant financial losses to the soybean production process globally. In China, *H. glycines* infection causes an average soybean yield loss of as high as 30%, and the figure even reaches 70% in some areas in Northeast China [1]. *H. glycines* infection causes similar yield losses in the USA [2]. Several methods such as conventional crossbreeding and transgenic breeding have been developed to confer resistance to soybean varieties against *H. glycines* [3]; however, more efficient methods must be developed to effectively combat this pest. Currently, the use of biocontrol agents is a potential pest-control method, which involves coating seeds with microorganisms to protect plants against microbes [4]. Seed treatment by introducing plant-parasitic nematodes (PPNs) has been extensively used to improve yield by parasitism, antibiosis, paralysis, and lytic enzymes [1,5,6,7,8,9,10]. Seed treatment indirectly induces different host defense pathways such as salicylic acid (SA), jasmonic acid (JA), auxin, cytokinin, and reactive oxygen species (ROS) signaling and chemical defense components through the synthesis of plant secondary metabolites or different enzymes to facilitate their parasitic successes in plants, which constitutes an advantage for the plant growth [11].

In the present study, we attempted to improve the root resistance of soybean against *H. glycines* through seed coating. *H. glycines* infection in soybean roots could be prevented by stimulating the roots with some active compounds isolated from microorganisms. These active compounds are typically evolutionarily conserved compounds such as saccharides, glycopeptides, lipids or lipopeptides, proteins, and low-molecular-weight metabolites [12,13], of which only protein-like or peptide-like plant immune elicitors can regulate plant resistance against nematodes [14,15]. The process of inducing plant immunity involves complete utilisation of the potential ability of plants to control disease occurrence. The regulation of plant defence and metabolic systems can induce the basic immune response and delay or reduce the occurrence and development of diseases [14,16].

The current research on the types of induced resistance responses to nematodes mainly focuses on two aspects. The first is the microscopic observation of the incompatible interactions between nematodes and their hosts [17], mainly microscopic observation of the developmental aspects of the infection site (syncytia and giant cells). The second is the identification and observation of rapid allergic reactions. Allergic necrosis is the defense mechanism of plants against a variety of pathogens. Its typical feature is oxidation, which leads to ROS production and the accumulation of phenylpropanoid compounds [18,19]. Acibenzolar S-methyl (ASM) is a plant immune elicitor for inducing resistance to PPNs through interference with a pectin lyase during the formation of giant cells affecting the development of nematodes. In addition, after using ASM for five days, the increase in β-1,3-glucanase activity was also an important factor in inducing resistance in plant roots [20,21,22]. The non-protein amino acid β-aminobutyric acid (BABA) inhibits the invasion of rice root-knot nematodes and delays the formation of giant cells and the development of nematodes depending on JA and ET pathways, while activating many basic defense responses, such as the accumulation of ROS, lignin formation, and callose deposition [23,24,25]. Sclareol is an antimicrobial and defense-related substance inhibiting the invasion of Meloidogyne incognita and inducing the expression of related genes such as ET biosynthesis and signal transduction pathways and phenylalanine metabolic pathways. In addition, sclareol activates mitogen-activated protein kinase (MAPK) MAPK3 and MAPK6, which are related to ET biosynthesis [26]. Silicon-induced defense reactions are related to phenylalanine lyase and lignin, peroxidase, and polyphenol oxidase. The silicon absorbed by plants can be quickly transferred to the roots to prevent the invasion of nematodes by activating the synthesis of phenols, lignin, cork and callosin, which are defense-related substances [23,26,27,28]. The induced resistance of plants is mainly regulated through the SA or JA/ET pathway. Therefore, exogenous application of SA, JA, or ET and other plant hormones and their analogues is a hot topic in the research of inducing resistance. Foliar spraying of epibrassinolide (BL) can inhibit the biosynthesis of BL in roots and negatively regulate the JA pathway to enhance rice resistance to root-knot nematodes [29,30,31]. However, the active compounds involved in conferring *H. glycines* resistance to soybean and the underlying mechanisms of seed treatment involved in stimulating the soybean immune system remain unknown.

In the present study, we aimed to (i) assess the biocontrol capability of Snef1650 towards *H. glycines* by performing pot and field tests; (ii) isolate and characterise scopoletin obtained from Snef1650; (iii) assess the direct in vitro antagonistic mechanism of scopoletin in juveniles of *H. glycines*; (iv) assess the biocontrol capability of scopoletin against *H. glycines* infection by performing field and pot experiments; and (v) confirm whether scopoletin inhibits syncytium formation through seed coating. The study can provide new insight into the utilisation of scopoletin as an anti-*H. glycines* biocontrol agent.

## 2. Results

### 2.1. Biocontrol of Heterodera Glycines by Snef1650 in the Pot and Field Tests

In the pot tests, the number of juveniles in the Snef1650-treated plants reduced by 45.96% compared with CK. The effect of Snef1650 on the development of plants was determined similarly. The root length, fresh root weight, and fresh shoot weight of plants treated with Snef1650 were significantly different from those of the control (Table 1).

In the field tests, the number of juveniles within roots of plants treated with Snef1650 reduced by 54.41% and 65.45% in 2016 and 2017, respectively. Meanwhile, the number of cysts per root in the plants treated with Snef1650 decreased by 60.64% and 57.19% in 2016 and 2017, respectively. Snef1650 treatment reduced the number of cysts in the soil by 70.08% and 66.79% in 2016 and 2017, respectively (Table 2).

In 2016 and 2017, the plant dried weight, the fresh soybean weight, and the root and shoot lengths in the Snef1650-treated plants differed from CK. Snef1650 treatment increased the root length by 21.06% and 17.4% in 2016 and 2017, respectively, and increased the shoot length by 6.36% and 3.52% in 2016 and 2017, respectively. In addition, Snef1650 treatment increased the fresh plant weight by 17.73% and 16.01% in 2016 and 2017, respectively. Snef1650 treatment also increased the dry plant weight by 14.44% and 15.31% in 2016 and 2017, respectively (Table 3). The treatment with Snef1650 significantly affected pod/seed quantities, plot yield, and 100-seed weight in 2016 and 2017. The treatment increased the number of pods by 8.99% and 6.91% in 2016 and 2017, respectively, and it increased the number of seeds by 2.6% and 2.51% in 2016 and 2017, respectively. The weight of 100 seeds was increased by 18.57% and 18.92% in the Snef1650-treated plants compared with that in the control. Meanwhile, the plot yield of the plants treated with Snef1650 increased by 5.78% and 5.83% in 2016 and 2017, respectively (Table 4).

### 2.2. Isolation and Denitrification of Scopoletin from Snef1650

**Figure 1:**
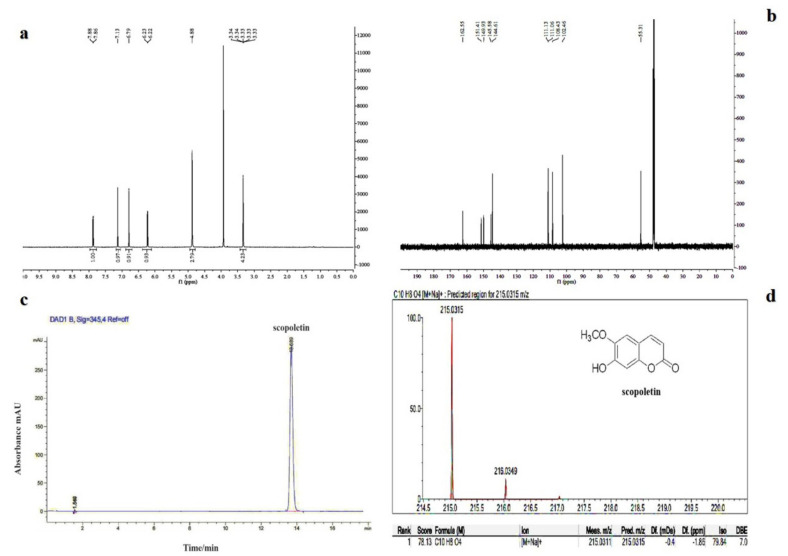
Effect of scopoletin on the juvenile mortality of Heterodera glycines.(**a**) HPLC performed for the structural analysis of scopoletin isolated from the Snef1650 fermentation broth; (**b**) HPLC–MS analysis of the structure of scopoletin obtained from the Snef1650 fermentation broth; (**c**) 1H NMR spectrum of scopoletin; (**d**) 13C NMR spectrum of scopoletin.

The metabolites from Snef1650 were separated and purified; their structures were determined through high-performance liquid chromatography (HPLC) in combination with nuclear magnetic resonance (NMR) and mass spectrometry (MS). HPLC detected the presence of pure compounds that had a single peak at 16.689 min (Figure 1a). HPLC–MS coupled with high-resolution electron ionisation–MS (EI–MS) detected the presence of purified compound with the molecular ion (M + Na) peak at (m/z) 215.0315. The molecular formula obtained was C10H8O4Na, and the calculated molecular weight was 215.0315 (Figure 1b). The active material structure was determined through NMR hydrogen spectroscopy and carbon spectroscopy (1H NMR and 13C NMR). The spectrum data of 1 H NMR (600 MHz, MeOH) were 7.87 (1H, d) and 7.13 (1H, s, H-8), which suggested that the two para-hydrogen signals of the tetra-substituted benzene ring, 6.79 (1H, s, H-4) and 6.22 (1H, d, H-3), were a pair of double-bond hydrogen signals, and the signal 3.33 (3H, s) was a methoxy signal (Figure 1c). The spectrum data of 13 C NMR (600 MHz, MeOH-d6) indicated that δC 162.55 was the α, β-unsaturated carbonyl signal; 151.41, 149.93, 145.58, 144.61, 111.13, 111.06, 108.43, and 102.46 were the double-bond carbon signals of the benzene ring; and 55.31 was the methoxy signal (Figure 1d).

### 2.3. Effect of Scopoletin on Juvenile Mortality of H. glycines

**Figure 2:**
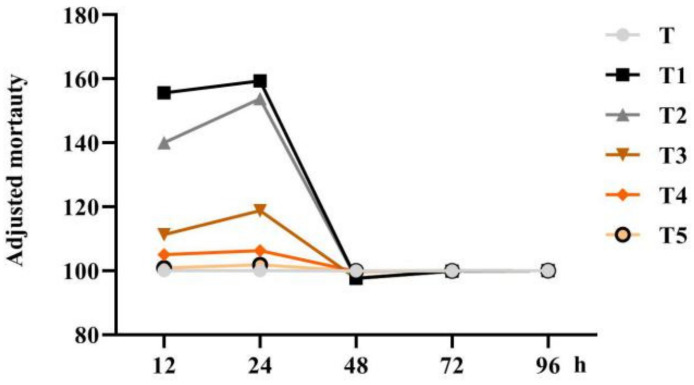
Biocontrol of *Heterodera glycines* by scopoletin in pot tests. Adjusted mortalities of second-stage juveniles exposed to different concentrations of scopoletin (S, T, T1, T2, T3, T4, and T5) for 12, 24, 48, 72, and 96 h (*n* = 6).

These results indicated that scopoletin exhibits the nematicidal activity. SCN second-stage juveniles (J2) were treated with different concentrations of scopoletin from high to low (T1, T2, T3, T4, and T5). High concentrations of scopoletin (T1 and T2) exhibited a significant effect on J2 poisoning, whereas the lower concentrations of scopoletin (T4 and T5) exhibited a negligible effect (Figure 2). The concentration T5 was used for the subsequent field experiment.

### 2.4. Impact of Scopoletin on Soybean Development and H. glycines Population in Pot Tests

**Figure 3:**
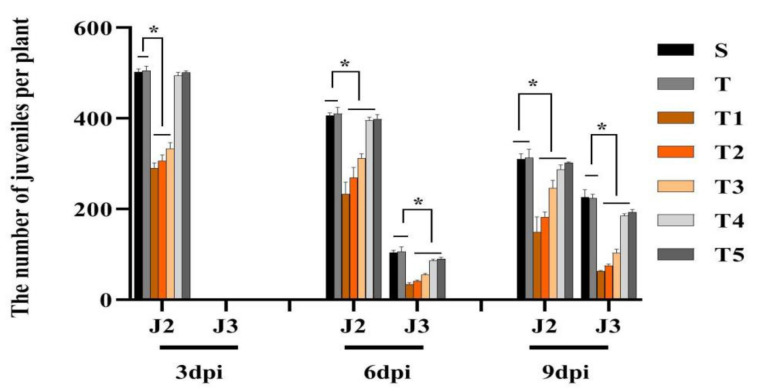
Effect of scopoletin on soybean growth in pot tests. The number of *H. glycines* at different developmental stages after treatment (S, T, T1, T2, T3, T4, and T5) at 3, 6, and 9 dpi. Scopoletin was dissolved in 1% methanol to obtain the following concentrations: T1: 81.00 µg/mL, T2: 40.50 µg/mL, T3: 20.25 µg/mL, T4: 4.05 µg/mL, and T5: 0.81 µg/mL; 1% methanol (T) dissolved in water (S) served as control. Asterisks indicate significant differences (* *p* < 0.05).

**Figure 4:**
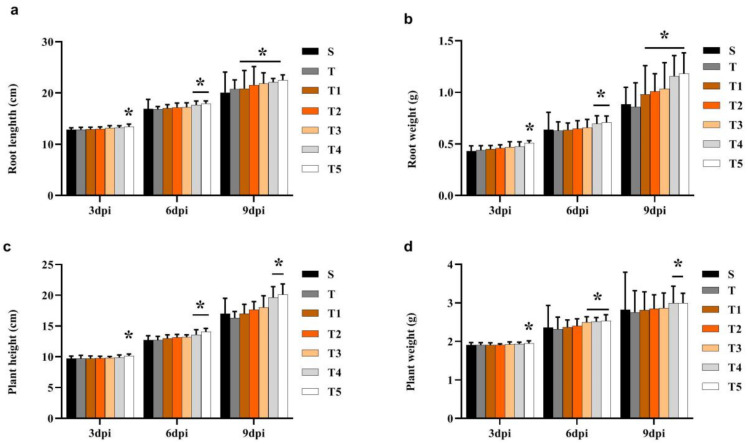
Histological evaluation of syncytium size in the roots at 9 dpi. The number of H. glycines at different developmental stages after treatment (S, T, T1, T2, T3, T4, and T5) at 3, 6, and 9 dpi; (**a**), soybean root length; (**b**), soybean plant height; (**c**), fresh root weight; (**d**), fresh shoot weight (*n* = 9). Scopoletin was dissolved in 1% methanol to prepare T1: 81.00 µg/mL, T2: 40.50 µg/mL, T3: 20.25 µg/mL, T4: 4.05 µg/mL, and T5: 0.81 µg/mL; and 1% methanol (T) dissolved in water (S) served as control. Asterisks indicate significant differences (* *p* < 0.05).

In the pot experiments, to determine the effect of scopoletin on soybean development, soybean seeds with SCN second-stage juvenile (J2) were coated with different concentrations of scopoletin (T1, T2, T3, T4, and T5). Scopoletin treatment delayed the SCN development. Meanwhile, all scopoletin concentrations significantly affected the quantity of J2 and J3 in soybean roots at 9 dpi (Figure 3). The concentration T1 had the maximum effect on the numbers of J2 and J3 in soybean roots at 9 dpi (Figure 4a). Meanwhile, the effect of scopoletin on plant development was also determined. The treatment with the low-concentration scopoletin (T4 and T5) treatment significantly affected the root length and fresh root/shoot weights at 6 dpi and 9 dpi (Figure 4b–d).

### 2.5. Scopoletin Inhibits Syncytium Formation through Seed Coating

**Figure 5:**
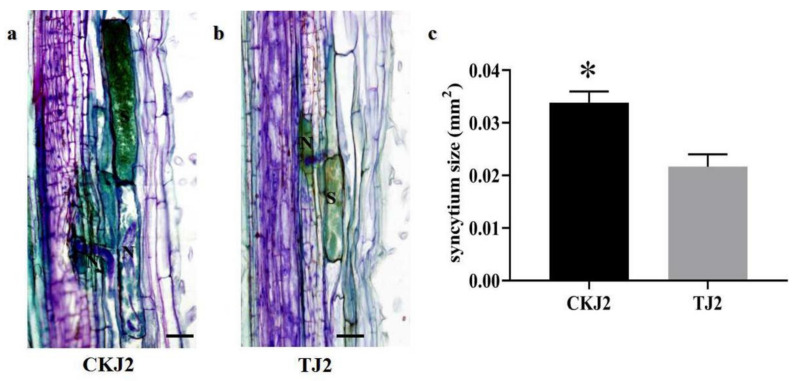
(**a**) SCN-induced JI17 root (CKJ2); SCN-induced syncytium was labelled as “S”; SCN was labelled as “N”(bar = 50 µm); (**b**) SCN-infected scopoletin-treated root (TJ2); SCN-induced syncytium was labelled as “S” (bar = 50 µm); (**c**) mean syncytium size based on TJ2 and CKJ2 values (bar; standard error (SE)). Five syncytia size values were used to perform the t-test, and *p* < 0.05 denotes significant differences between TJ2 and CKJ2.

The establishment and development of syncytium can be affected by scopoletin treatment. We determined the syncytium size induced by SCN under T and CK treatments at 9 dpi. The mean syncytium size was 0.034 mm^2^, and the average syncytium size was 0.022 mm^2^ in T after CK treatment. Scopoletin treatment significantly decreased the average syncytium size by 35.29% compared with CK treatment at 9 dpi (Figure 5).

### 2.6. Biocontrol of H. glycines by Scopoletin in Field Experiments

In the field tests, scopoletin treatment decreased the number of juveniles inside the root by 49.45% and 43.7% in 2017 and 2018, respectively. Scopoletin treatment also decreased the number of cysts per root by 55.9% and 53.75% in 2017 and 2018, respectively. Additionally, this treatment decreased the number of cysts in the soil by 64.06% and 61.55% in 2017 and 2018, respectively (Table 5).

The scopoletin treatment significantly affected soybean root/shoot lengths and plant fresh/dry weights in either year compared with those in the control. Scopoletin treatment increased the shoot length by 19.27% and 17.85% in 2017 and 2018, respectively. The treatment increased root length by 2.9% and 3.1% in 2017 and 2018, respectively. Additionally, the treatment increased the fresh weight of plants by 17.15% and 13.47% in 2017 and 2018, respectively. The treatment also increased the dry plant weight by 19.1% and 21.11% in 2017 and 2018, respectively (Table 6).

The scopoletin treatment significantly increased the number of pods, seeds, the 100-seed weight, and the plot yield in 2017 and 2018. The treatment significantly increased the number of pods by 8.99% and 6.91% in 2017 and 2018, respectively, and the number of seeds by 2.54% and 2.45% in 2017 and 2018, respectively. In addition, the scopoletin treatment increased the 100-seed weight by 15.66% and 15.91% in 2017 and 2018, respectively. Meanwhile, the treatment increased the plot yield by 5.33% and 5.64% in 2017 and 2018, respectively (Table 7).

## 3. Discussion

In the present study, Snef1650, a potential seed treatment agent for controlling nematodes, was used to improve soybean resistance against *H. glycines*. Soybean seeds of susceptible cultivars treated with the Snef1650 fermentation liquid demonstrated increased SCN tolerance and a boost in growth and yield. Studies have demonstrated that microorganism-treated seeds produce nematicides during root development and resist infection due to different parasitic nematodes through systemic or local induction of endogenous hormones and plant defense-related proteins in the plant immune system [32,33,34,35,36,37].

Scoploletin is a type of plant secondary metabolite produced by the phenylalanine metabolism pathway, which is abundantly present in plants and involved in the plant defense response to stress [38]. Although scopoletin is found in more than 110,000 plants belonging to several families exhibiting direct or indirect resistance against other plant diseases [10,15,39,40,41,42,43,44,45], it had directly detected nematocidal activity and has not yet indirectly induced resistance to nematode in microorganisms. In this study, scoploletin was obtained for the first time through separation and purification of active substances in the fermentation broth of Snef1650. This metabolite was found to be a potent compound and exhibited efficient agent and nematicidal activity in pot experiments and field trials. The presence of scopoletin in microorganisms could be explained by the similarities in enzymes, substrates, and pathways for scopoletin synthesis across various plants and microorganisms. Though the pathways for the biosynthesis of scoploletin exist in different microbes, its extracts outside the plant kingdom with the specific objective of its application to plants against nematodes were explored for the first time in this work. Additionally, the horizontal gene transfer due to long-term coexistence or interaction between microorganisms and plants could be responsible for plant immunity against PPNs and increased soybean yield [46,47,48]. It will be interesting to explore the effect of scopoletin on the root zone dwelling microbes and insects (both pests and friendly ones) in future studies. Additionally, scopoletin might be used as a selection marker for the offspring in breeding *H. glycines*-resistant soybean and the possible genetic players of scopoletin metabolism pathway in soybean that might be manipulated for breeding *H. glycines*-resistant soybean.

The syncytium is a nurse attendant cell that is positioned inside the soybean roots by *H. glycines*. *H. glycines*’ survival depends on the development and maintenance of the syncytium. Under nematode stress conditions, resistant soybean cultivars confine the arrangement and advancement of syncytia through several mechanisms during the early phases of soybean–SCN interactions [49,50,51,52,53,54,55,56]. Nevertheless, the scopoletin-coating effect on suppressing the development of syncytia in susceptible soybean cultivars has been unclear. Scopoletin treatment slows down syncytium formation, leading to the inhibition of nematode development.

To summarise, both field and pot experiments in this study indicated that soybean seeds treated with scoploletin, obtained from the Snef1650 fermentation broth, exhibited remarkably decreased numbers of *H. glycines* juveniles and cysts. Scopoletin decreased nematode development in roots and reduced J2 activity in vitro. Additionally, scopoletin significantly promoted the growth of soybean. These results indicate that scopoletin from *Penicillium janthinellum* Snef1650 has the potential for controlling *H. glycines* infection.

## 4. Materials and Methods

### 4.1. Fungal Strain and Culture Conditions

Snef1650 (*Penicillium janthinellum*) was chosen from 4600 fungal strains detached from rhizosphere soil test samples, harvested from various regions of China, because of its ability to inhibit *H. glycines*. Snef1650 was obtained from Northern Nematode Research Institute (NINC), Shenyang Agricultural University, China (CGMCC No. 10487; Patent No.: CN201510607223.9 and CN201810553440.8). Snef1650 was cultured in potato dextrose agar (PDA) at 25 °C for 7 days to prepare the seed-coating agent. The Snef1650 culture plates were punched using a 5-mm diameter punch, and the strain was inoculated into a 50-mL flask for performing a sterilisation test. In an Erlenmeyer flask of liquid fermentation medium, the culture was kept in a shaker incubator (120 rpm) at 25 °C for 8 days. The cultured fermentation broth was filtered through a qualitative filter paper to remove the dense mycelium and then placed in a refrigerator at 4 °C for future use.

### 4.2. Preparation of Nematodes

*H. glycines* was isolated from the soil of the NINC experimental field. First, the soil was passed through a set of 420- and 250-μm sieves to collect cysts and was sterilised with 0.5% NaOCl. The surface was sterilised for 3 min, washed thrice with sterile distilled water, and placed in a 25 °C incubator to incubate the second-stage juvenile (J2) prior to collecting J2 (2000 J2 mL^−1^) for inoculation.

### 4.3. Biocontrol of H. glycines by Snef1650 in Pot Tests

Jidou 17, a soybean variety susceptible to *H. glycines*, was used in all the tests (provided by researcher Zhang Mengchen, Hebei Academy of Agricultural Sciences, China, No. National Examination Bean 2013010). Snef1650 culture (10^9^ CFU/mL) was used for the uniform coating of seeds at 70:1 (*w:v*), according to the method described by Jing. Z [57]. At 9 days post-inoculation (dpi), we calculated juvenile quantity within roots according to acid fuchsin staining proposed by Byrd and colleagues [6]. The amount of juveniles inside the roots, fresh root/shoot weights, and root length were recorded. Ten replicates were set, and this experiment was performed in duplicates.

### 4.4. Biocontrol of Heterodera Glycines by Snef1650 in Field Tests

Snef1650 fermentation broth was used to coat soybean seeds in May 2016 and 2017 at NINC of Shenyang Agricultural University (123.65bE, 42.32bN). Field trials based on the well-recognised serious SCN infection (>50 cysts/100 mL soil) were performed in the naturally occurring test fields, according to the method described by Jing. Z [57]. Fresh weight, root/shoot lengths of the above seedlings, juvenile quantity within roots, cyst number on roots, cysts per 100 mL, dry weight of the seedlings, seed/pod quantities in each plant, yield per plot, and 100-seed weight were recorded.

### 4.5. Isolation, Purification, and Structural Analysis of Scopoletin

The Snef1650 fermentation broth was separated and purified through organic solvent extraction, silica gel column, and semi-preparative HPLC, according to the method described by Xing zhifu [58]. Snef1650 was cultured on PDA at 25 °C for 7 days, and three pieces of Snef1650 were punched with a 5-mm diameter punch and inoculated with 50 mL of sterile Chagas liquid fermentation medium. The culture was kept in a shaker incubator (120 rpm) at 25 °C. Day-8 culture was used as a seed fermentation broth and was added to a fully automatic mechanically stirred stainless steel fermentation tank for large-scale preparation of 25-L Chac liquid fermentation broth. The Snef1650 fermentation broth was centrifuged in a high-speed centrifuge, evaporated and concentrated, and then mixed with ethanol (1:1, *v/v*). The concentrates were separated using a silica gel column through gradient elution with dichloromethane:methanol. A total of 82 tubes were collected. Thin-layer chromatography was performed to combine similar fractions of the concentrates, methane/methanol 90:10 (*v/v*), and then they were freeze-dried. Using hydroxamic acid iron reagent, we determined the fourth component as coumarin and further isolated different components and purified them through semi-preparative HPLC under the following chromatographic conditions: semi-preparative chromatography column AQ-C18; mobile phases consisting of 0.1% formic acid aqueous solution (A) and methanol (B) under gradient elution (0–10 min, 85%–66% A; 10–20 min, 66% A); 1.5 mL/min flow rate; 25 °C temperature; 345 nm detection wavelength; and 100 μL injection volume. The corresponding absorption peaks were evaluated and interpreted to obtain compound 1.

To analyse the structure of compound 1 in the purified metabolites of Snef1650, HPLC–MS, and NMR spectroscopy were performed. The chromatographic column for HPLC–MS detection was the Agilent Extend C18 column; other conditions were similar to those for semi-preparative HPLC. The mass spectrometric analysis was performed using AB Sciex QTRAPTM 3200 series quadrupole linear ion trap mass spectrometer (Applied Biosystems, USA). Mass spectrometric analysis conditions were maintained as described by Xing Zhifu [58]. The structure of compound 1 was detected using NMR spectroscopy (^1^H NMR and ^13^C NMR) (Brook 600 MHz, Karlsruhe, Germany). NMR conditions included a deuterated reagent to dissolve the sample, and the scanned ranges were −2–16 ppm and 0–220 ppm for ^1^H NMR and ^13^C NMR, respectively, at 25 °C.

### 4.6. Effect of Scopoletin on Juveline Mortality In Vitro

An experiment was performed to test scopoletin’s nematicidal properties by adding the second instar larval suspension of the SCN (200 J2/50 L) to the 1-mL sample solution in a 35-mm Petri dish. Nematodes were treated with seven solutions (S, T, T1, T2, T3, T4, and T5). After cultivating the Petri dish containing nematodes in a constant-temperature incubator (25 ± 2 °C) for 12, 24, 48, 72, and 96 h, the second-stage juveniles of live and dead SCNs were counted under an optical microscope (*n* = 6). The adjusted mortality was calculated according to the following formula: adjusted mortality = (treatment mortality − control mortality)/control mortality × 100 + 100.

Scopoletin was dissolved in 1% methanol to prepare T1 (81.00 μg/mL), T2 (40.50 μg/mL), T3 (20.25 μg/mL), T4 (4.05 μg/mL), and T5 (0.81 μg/mL). In addition, 1% methanol (T) in water (S) served as a control (CK). Fine needles were used to detect deformed, immobile, or stationary juveniles, which were considered dead.

### 4.7. Biocontrol of H. glycines by Scopoletin in Pot Tests

Seeds were coated with scopoletin in a proportion of approximately 70:1 (*w:v*). At 3, 6, and 9 dpi, the amount of juveniles inside the roots, root length, fresh root weight, and fresh shoot weight were recorded. The test design reference was 4.3. The optimal concentration of scopoletin was used for field trials.

### 4.8. Biocontrol of H. glycines by Scopoletin in Field Tests

The optimal concentration of scopoletin was used in pot tests for the field test. Soybean seeds were coated with scopoletin in May 2017 and 2018 at NINC of Shenyang Agricultural University. Scoploletin was applied to soybean seeds in a 70:1 (g/mL) ratio. The test design reference was 4.4. Fresh weight, root/shoot lengths of the above seedlings, juvenile quantity within roots and cyst quantity on roots, cysts per 100 mL, dry weight of the seedlings, pod/seed quantities in each plant, yield per plot, and 100-seed weight were recorded.

### 4.9. Histological Observation

For observing histological alterations in TJ2 and CKJ2 at 9 dpi, root samples were cut in 1-cm cubes prior to fixation with Carnoy’s fix solution for measuring the syncytia, according to the method described by Lei, P [55].

### 4.10. Data Analysis

SPSS17.0 (SPSS, Inc., Chicago, IL, USA) and Microsoft Office Excel 2010 were used for statistical analyses. Analysis of variance (ANOVA), *t*-test, and Duncan’s multirange test were used to analyse differences among various treatments. A *p* value of <0.05 was considered to denote statistical significance.

## 5. Conclusions

SCN (*Heterodera glycines* Ichinohe) causes considerable damage to soybean cultivations globally. Treatment of soybean seeds with *Penicillium janthinellum* Snef1650 remarkably decreased *H. glycines* juvenile and cyst numbers in field and pot tests. Scoploletin was isolated and purified from the Snef1650 fermentation broth that decreased nematode development in roots and reduced J2 activity in vitro. Scopoletin also promoted soybean growth significantly. These results suggest that scopoletin from *Penicillium janthinellum* Snef1650 has the potential for controlling *H. glycines* infection. The present study may provide a theoretical foundation for a biocontrol agent for better control of SCN. However, further investigations are desired to explore the biocontrol activity of *Penicillium janthinellum* Snef1650 against different nematodes’ species and host plants.

## Figures and Tables

**Table 1:** Biocontrol of *Heterodera glycines* and effect of Snef1650 on soybean growth in pot tests.

Treatment	Juveniles Per Root ^a^	Percentages of Reduction	Root Length (cm) ^a^	Fresh Shoot Weight (g) ^a^	Fresh Root Weight (g) ^a^
CK	369.85 ± 24.35	-	16.85 ± 0.61	2.59 ± 0.05	0.8 ± 0.05
Snef1650	199.85 ± 7.35 *	45.96%	20.09 ± 0.56 *	3.00 ± 0.06 *	1.3 ± 0.06 *

Results are expressed as mean ± SE (*n* = 20). * stands for significant significance upon Student’s *t*-test (*p* < 0.05). ^a^: Data are tested by the nonparametric Mann–Whitney test (*p* < 0.05).

**Table 2:** Biocontrol of *Heterodera glycines* by Snef1650 in pot tests in 2016 and 2017.

	Treatment	Juveniles Per Root ^a,†^	Percentages of Juveniles Reduction	Cysts Per Root ^a,‡^	Percentages of Reduction	Cysts Per 100 mL Soil ^a,§^	Percentages of Reduction
2016	CK	24.48 ± 3.32	-	78.74 ± 6.3	-	128 ± 3.59	-
	Snef1650	11.16 ± 1.82 *	54.41%	30.99 ± 6.09 *	60.64%	38.3 ± 2.5 *	70.08%
2017	CK	28.48 ± 3.18	-	82.57 ± 9.09	-	157.2 ± 2.97	-
	Snef1650	9.84 ± 1.28 *	65.45%	35.35 ± 5.84 *	57.19%	52.2 ± 4.42 *	66.79%

Results are expressed as mean ± SE (^†^: *n* = 25; ^‡^: *n* = 125; ^§^: *n* = 10). * stands for significant significance upon Student’s *t*-test (*p* < 0.05). ^a^: Data are tested by the nonparametric Mann–Whitney test (*p* < 0.05).

**Table 3:** Effect of Snef1650 on soybean plant growth in field tests.

	Treatment	Shoot Length (cm) ^a^	Percentages of Increase	Root Length (cm) ^a^	Percentages of Increase	Fresh Plant Weight (g) ^a^	Percentages of Increase	Dry Plant Weight (g) ^a,†^	Percentages of Increase
2016	CK	9.52 ± 0.29	-	15.91 ± 0.61	-	2.99 ± 0.14	-	0.77 ± 0.09	-
	Snef1650	12.06 ± 0.55	21.06%	16.99 ± 0.54	6.36%	3.52 ± 0.18	17.73%	0.9 ± 0.22	14.44%
2017	CK	9.49 ± 0.32	-	15.93 ± 0.57	-	3.06 ± 0.14	-	0.83 ± 0.1	-
	Snef1650	11.49 ± 0.29	17.4%	16.49 ± 0.27	3.52%	3.55 ± 0.21	16.01%	0.98 ± 0.11	15.31%

Results are expressed in the form of mean ± SE (*n* = 50; ^†^: *n* = 20). * stands for significant significance upon Student’s *t*-test (*p* < 0.05). ^a^: Data are tested by the nonparametric Mann–Whitney test (*p* < 0.05).

**Table 4:** Effect of Snef1650 on soybean agronomic traits in field tests.

	Treatment	Pods Per Plant ^a^	Percentages of Increase	Seeds Per Plant ^a^	Percentages of Increase	100-Seed Weight (g) ^a,†^	Percentages of Increase	Yield Per Plot (kg) ^2 a,‡^	Percentages of Increase
2016	CK	41.62 ± 1.9	-	92.58 ± 5.15	-	15.51 ± 0.41	-	20.96 ± 0.03	-
	Snef1650	45.73 ± 4.27 *	8.99%	94.99 ± 5.22 *	2.6%	18.39 ± 0.37 *	18.57%	22.17 ± 0.03 *	5.78%
2017	CK	41.34 ± 2.34	-	92.50 ± 5.08	-	15.54 ± 0.27	-	20.94 ± 0.03	-
	Snef1650	44.41 ± 2.61 *	6.91%	94.82 ± 4.9 *	2.51%	18.48 ± 0.28 *	18.92%	22.16 ± 0.03 *	5.83%

Results are expressed in the form of mean ± SE (*n* = 50; ^†^: *n* = 20; ^‡^ *n* = 5). * stands for significant significance upon Student’s *t*-test (*p* < 0.05). ^a^: Data are tested by the nonparametric Mann–Whitney test (*p* < 0.05).

**Table 5:** Biocontrol of *Heterodera glycines* by scopoletin in field tests.

	Treatment	Juveniles Per Root ^a,†^	Percentages of Reduction	Cysts Per Root ^a,‡^	Percentages of Reduction	Cysts Per 100 mL Soil ^a,§^	Percentages of Reduction
2017	CK	21.68 ± 2.58	-	90.67 ± 4.92	-	167.5 ± 5.84	-
	Scopoletin	10.96 ± 1.77 *	49.45%	39.98 ± 4.69 *	55.9%	60.2 ± 1.69 *	64.06%
2018	CK	19.68 ± 3.7	-	92.43 ± 5.39	-	181.8 ± 3.08	-
	Scopoletin	11.08 ± 2.18 *	43.7%	42.75 ± 3.68 *	53.75%	69.9 ± 3 *	61.55%

Results are expressed in the form of mean ± SE (^†^: *n* = 25; ^‡^: *n* = 125; ^§^: *n* = 10). * stands for significant significance upon Student’s *t*-test (*p* < 0.05). ^a^: Data are tested by the nonparametric Mann–Whitney test (*p* < 0.05).

**Table 6:** Effect of scopoletin on soybean plant growth in field tests.

	Treatment	Shoot Length (cm) ^a^	Percentages of Increase	Root Length (cm) ^a^	Percentages of Increase	Fresh Plant Weight (g) ^a^	Percentages of Increase	Dry Plant Weight (g) ^a,†^	Percentages of Increase
2017	CK	8.84 ± 0.59	-	15.09 ± 0.44	-	3.44 ± 0.35	-	0.72 ± 0.11	-
	Scopoletin	10.95 ± 0.53 *	19.27%	15.54 ± 0.3 *	2.9%	4.03 ± 0.54 *	17.15%	0.89 ± 0.1 *	19.1%
2018	CK	9.02 ± 0.56	-	15.01 ± 0.57	-	3.47 ± 0.28	-	0.71 ± 0.12	-
	Scopoletin	10.98 ± 0.58 *	17.85%	15.49 ± 0.32 *	3.1%	4.01 ± 0.55 *	13.47%	0.9 ± 0.1 *	21.11%

Results are expressed in the form of mean ± SE (*n* = 50; ^†^: *n* = 20). * stands for significant significance upon Student’s *t*-test (*p* < 0.05). ^a^: Data are tested by the nonparametric Mann–Whitney test (*p* < 0.05).

**Table 7:** Effect of scopoletin on soybean agronomic traits in field tests.

	Treatment	Pods Per Plant ^a^	Percentages of Increase	Seeds Per Plant ^a^	Percentages of Increase	100-Seed Weight (g) ^a^	Percentages of Increase	Yield Per Plot (kg) ^2a^	Percentages of Increase
2017	CK	41.62 ± 1.9	-	92.58 ± 5.15	-	15.51 ± 0.41	-	20.97 ± 0.04	-
	Scopoletin	45.73 ± 4.27 *	8.99%	94.99 ± 5.22 *	2.54%	18.39 ± 0.37 *	15.66%	22.15 ± 0.03 *	5.33%
2018	CK	41.34 ± 2.34	-	92.50 ± 5.08	-	15.54 ± 0.27	-	20.91 ± 0.03	-
	Scopoletin	44.41 ± 2.61 *	6.91%	94.82 ± 4.9 *	2.45%	18.48 ± 0.28 *	15.91%	22.16 ± 0.04 *	5.64%

Results are expressed in the form of mean ± SE (*n* = 50; ^†^: *n* = 20; ^‡^: *n* = 5). * stands for significant significance upon Student’s *t*-test (*p* < 0.05). ^a^: Data are tested by the nonparametric Mann–Whitney test (*p* < 0.05).

## Data Availability

The data presented in this study are available in the main text.
